# Profile-guided Hybrid Approach for block-wise missing data handling in multi-omics: a breast cancer case study

**DOI:** 10.1186/s13040-026-00530-8

**Published:** 2026-03-20

**Authors:** Esraa Hamdi Abdelaziz, Eman Amin, Rasha Ismail, Mai Mabrouk

**Affiliations:** 1 Faculty of Computer and Information Sciences, Ain Shams University, Cairo, Egypt; 2 Center for Informatics Science (CIS), School of Information Technology and Computer Science, Nile University, Giza, Egypt

**Keywords:** Block-wise missingness, Multi-omics integration, Imputation, Available-case modeling, Deep learning, Hybrid Approach, Multi-omics data mining

## Abstract

**Background:**

Block-wise missingness is a common challenge in multi-omics data, hindering the development of robust and generalizable machine learning models, as real-world cohorts rarely contain complete omic profiles. Many current methods either discard incomplete samples, use available-case models that need retraining when faced with new missingness patterns, or depend on full-dataset imputation, which can risk biological integrity and model stability.

**Methods:**

Using a complete four-omics breast cancer dataset (705 patients, 1,937 features), up to 60% block-wise missingness was simulated across five clinically relevant scenarios and used to compare four strategies for handling missing data: an Imputation-Based model, Dynamic and Exhaustive Available-Case approaches, and the proposed Hybrid Approach that combines profile-guided modeling with selective, test-time imputation. Performance was evaluated using accuracy, F1 score, balanced accuracy, inference time, and variability across 15 random seeds, with significance assessed using the Wilcoxon signed-rank test.

**Results:**

The Hybrid Approach consistently achieved the strongest and most stable performance. Relative to the complete-data baseline, it reached an average accuracy of 103.7%, F1 score of 123.3%, and balanced accuracy of 104.8%, outperforming the Imputation-Based method and matching or exceeding both Dynamic and Exhaustive Available-Case strategies. Statistical testing confirmed that these improvements were significant. The method also demonstrated fast and predictable inference (~ 2 s) and an average total runtime of ~ 49 s per configuration—nearly three times faster than the Exhaustive approach (~ 124 s)—while maintaining high reproducibility and low variance across seeds, a key indicator of computational stability.

**Conclusion:**

By selectively combining lightweight imputation with profile-specific modeling, the Hybrid Approach provides a computationally efficient and statistically robust solution for block-wise missing data. This framework offers a generalizable strategy for multi-omics data mining, and lays the foundation for future systems incorporating cross-profile learning and advanced imputation.

## Background

The integration of multi-omics data has emerged as a powerful approach for uncovering complex biological mechanisms and improving disease prediction, diagnosis, and treatment strategies. Given the inherently complex and hierarchical nature of multi-omics data, advanced computational methods, including network-based analyses, are increasingly necessary to encode and interpret the relationships between omics layers [1]. Furthermore, recent reviews highlight the growing reliance on deep learning and machine learning to effectively integrate these high-dimensional datasets while emphasizing that handling incomplete or missing modalities remains an unresolved challenge for multi-omics modeling [2]. However, although these models show great promise, they cannot be reliably deployed unless they remain robust when certain omics are incomplete.

Despite the growing availability of multi-omics datasets, missing data remains a critical challenge since patients are usually represented for some but not all omics layers in multi-omics datasets due to cost constraints, technical limitations, or experimental considerations [3]. A very common pattern of missingness in multi-omics datasets is block-wise missingness, which refers to a structured pattern of missing data where an entire block (omic) of related features is missing together, not just random individual values [4]. For example, a multi-omics dataset might include genomics, transcriptomics, and proteomics data, but some samples or subjects may be missing one or more of these data types. In real-world scenarios, it is common for certain omics layers to be entirely missing for subsets of samples, leading to difficulties in integration and downstream analysis.

According to a recent comprehensive review on missing multi-omics data [5], existing methods for handling block-wise missing multi-omics data can be broadly classified into three main categories. First, naive approaches which include discarding incomplete samples or training models on a single omics block shared across datasets. However, these strategies are very impractical especially if most patients have a missing omic or more which would lead to heavy information loss. Second, Imputation-Based approaches, which aim to reconstruct missing values using general techniques. These approaches are effective for handling randomly scattered missing values. However, in block-wise missingness, where large portions of entire feature groups are missing, these approaches can lead to inaccurate estimations, which may distort the data distribution and harm model performance [6]. Third, available-case approaches avoid imputation altogether by training separate models based on the missingness structure in the datasets. Although these approaches do not need to impute missing data and therefore preserve biological integrity, they often must be retrained for each different missingness pattern in the test data, which may be computationally expensive in practice.

In this paper, we propose a Hybrid Approach for patient-level outcome prediction and prognostic modeling under block-wise missing multi-omics data, combining elements of available-case modeling and imputation-based strategies. Our approach constructs specialized prediction models based on the missingness patterns observed in the training dataset, allowing maximal information to be extracted while preserving the biological integrity of the data by minimizing reliance on imputation. Imputation is applied only at the testing stage, and solely to align a given test sample with one or more of the pre-trained models. This design eliminates the need to retrain models when encountering new missingness patterns in the test data, offering a flexible solution for block-wise missingness in multi-omics datasets and improving the robustness of downstream predictive modeling.

To evaluate our approach, we designed an experiment to compare it against three representative approaches for handling missing data. These include a full imputation approach and two available-case approaches: one that trains models only for the missing patterns present in the dataset, and another that builds models for all possible missingness patterns. The proposed Hybrid Approach is then assessed alongside these approaches to demonstrate its effectiveness.

This study introduces a novel Hybrid Framework, demonstrating the feasibility and significant computational advantages of combining available-case approaches with selective imputation. Since the current implementation prioritizes conceptual clarity over optimization, relatively simple models are used and a basic approach to available-case data categorization. However, we acknowledge that incorporating more advanced modeling techniques, refined imputation methods, and shared learning across profiles could further enhance predictive performance.

The remainder of this paper is organized as follows: Sect. “Related work” reviews related work on multi-omics missing data handling. Section “Materials and Methods” describes the datasets, experimental design, baseline methods, and our proposed approach. Section “Results” presents the comparative results across various missingness scenarios. Section “Discussion” discusses the findings, limitations, and future directions. Finally, Sect. “Conclusion” concludes the paper.

## Related work

Block-wise missing data remains a major challenge in multi-omics integration. Although many predictive modeling strategies have been developed, only a few are specifically designed to offer explicit support for this type of missingness [7]. In this section, we summarize recent studies that address block-wise missing data either through Imputation-Based techniques or available-case approaches.

Many conventional Imputation-Based approaches are not optimized for block-wise missing data. However, one popular approach is the TOBMI kNN [8] method which was proposed to impute missing RNA-seq data using information from the DNA methylation data. It uses DNA methylation data to identify k “donor” samples using Mahalanobis distance. Then, it uses a weighted average of these donors to impute missing gene expression values. Although effective for cross-omics reconstruction, TOBMI does not explicitly address cases where entire omics blocks are missing.

Xue and Qu [9] handle block-wise missing data by grouping samples with the same missing patterns and performing multiple imputations within each group. The models are trained on each imputed dataset and the results are combined for better predictions. On the other hand, the Priority-LASSO-impute method [10] handles missing data by giving priority to different omics blocks based on their importance. It builds prediction models step-by-step, starting with the most important block. Instead of directly filling in missing data, it estimates missing parts based on information from higher-priority blocks.

A clear limitation of imputation approaches is that increased sophistication, especially when performing multiple imputation, often leads to significantly higher computational demands [5]. Moreover, because imputation techniques rely on model-based or random predictions that could vary between analyses, reproducibility might decrease and the biological integrity in the data may also be compromised. Studies have shown that the choice of imputation strategy can significantly influence downstream results and model stability [11], highlighting the need for cautious application of imputation or the use of alternative strategies [12].

To avoid imputation, the concept of profiles where datasets are partitioned according to the availability of omics, was introduced. For example, if three omics are available A, B, and C, then one group could have complete data of A and B, the other would have complete data of B and C and so on. An early study [13] proposed an ensemble-based classification framework to handle block-wise missing features by training classifiers on subsets of available modalities and combining their outputs via weighted aggregation.

Similarly, the iMSF model [14] splits the data into separate, non-overlapping groups based on which sources are available and trains a different model for each group. It doesn’t allow sharing between groups, in contrast to the improved version, the iSFS model [15], which allows overlapping sources across groups, and learns shared feature weights. The method assigns weights to each omic according to its relevance to the prediction task. It prioritizes the most informative sources by giving them higher weights, while sources with little or no contribution receive zero weight and are effectively excluded from the model.

To classify asthma outcomes in children, authors in [16] train individual models for each omics block independently, using all samples containing that block. Predictions for a test sample are obtained by combining outputs from the models corresponding to the blocks present, weighted according to cross-validated AUC values.

Another approach using the profile concept is presented in [17] where samples are categorized into profiles and a unified global model is trained by learning shared source-specific parameters and profile-specific weights, allowing efficient use of all available data without imputation. This framework was extended in [18] for multi-class classification support in addition to the previously supported binary and continuous response types.

By directly addressing missing data without imputation, previously mentioned methods provide an advantage and preserve the biological integrity of the data. However, they often face a choice between retraining whenever an unseen missingness pattern appears, pretraining every possible block combination, which is computationally expensive [5] or simply ignoring the missing omic at test time, thereby discarding information that the already-trained models could still exploit.

A separate but related class of methods leverages advanced machine learning for either sophisticated imputation or latent-factor-based integration. For instance, deep learning–based imputers such as GAIN and Deep Matrix Factorization (DeepMF) aim to reconstruct missing values with high fidelity by modeling complex data distributions [19, 20]. Similarly, integration frameworks like Multi-Omics Factor Analysis (MOFA2) and CustOmics identify shared and distinct sources of variation across omics layers, often by projecting data into a lower-dimensional latent space [21, 22]. While these methods are powerful for data completion or representation learning, they rely on a single unified prediction or reconstruction model applied to fully imputed or latent data representations. hey are not designed to address test-time block-wise missingness or to leverage profile-specific ensembles for prediction.

In contrast, our Hybrid Approach maintains biological data integrity by combining the profile concept with selective, lightweight imputation. Separate models are trained for each observed missingness pattern, preserving the authenticity of available data without unnecessary completion. At test time, when a novel missing pattern appears, minimal imputation is applied solely to align the incomplete sample with one or more pre-trained available-case models. This routing mechanism enables accurate predictions across unseen configurations while avoiding full retraining, ensuring both computational efficiency and biological validity.

## Materials and methods

### Dataset

Most existing studies on block-wise missing data focus on datasets containing three-omics. To evaluate the applicability of our approach to more complex multi-omics scenarios, a dataset containing four-omics layers was selected: somatic mutation (mu), copy number variation (cn), RNA sequencing (rs), and phospho-protein expression (pp). The dataset includes 705 breast cancer patients (611 alive, 94 deceased) and 1,937 features distributed across four omics layers: RNA sequencing (604 features), copy number variation (860 features), somatic mutation (249 features), and phospho-protein expression (223 features), with a target variable indicating vital status (survived or died). Survival is inherently a time-to-event outcome; however, for this methodological comparison, we follow standard practice in bioinformatics by using the binary outcome provided in the dataset to enable consistent evaluation across missing-data handling strategies. The original dataset is complete with no missing values, allowing us to simulate controlled missingness scenarios. The dataset used in this study was obtained from a publicly available source [23].

The dataset was split into training and testing subsets using a stratified sampling method that preserved the original class distribution. 5% of the total patient group was set aside for external validation, ensuring proportional representation of outcome classes and enabling fair evaluation of predictive performance under clinically relevant class imbalance. Controlled patterns of missing data were then introduced separately into the training and testing subsets. Additionally, unchanged versions of the training and testing sets were kept as complete reference datasets for control comparison.

Because missing-data handling methods may interact differently with high-dimensional omics features, using a fully complete dataset allowed methodological effects to be isolated without introducing confounding sources of missingness.

### Missing data scenarios

**Fig. 1:**
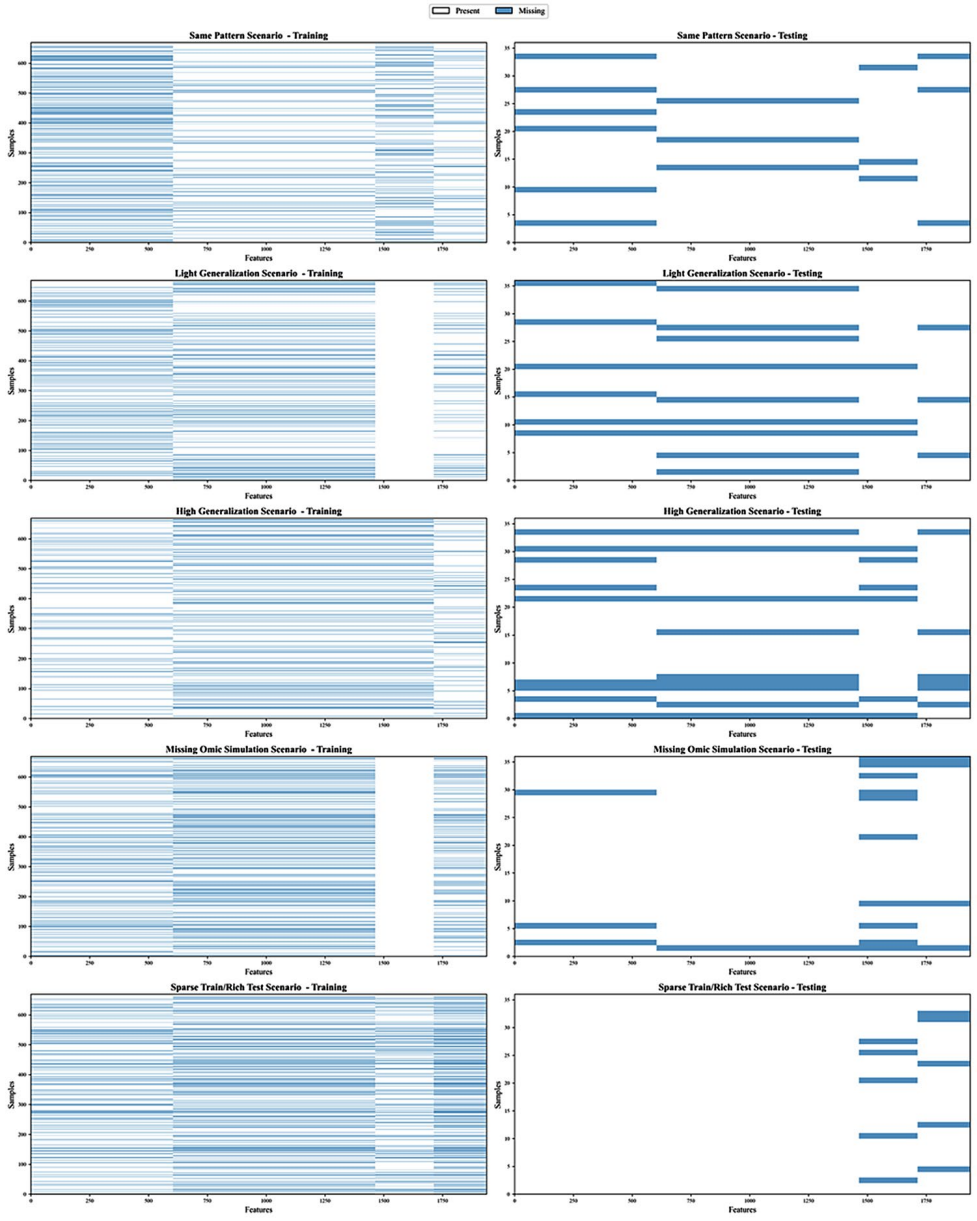
Missingness heatmaps for training and testing sets across five scenarios. Blue = missing; white = observed. Rows: samples; columns: features

For the experiments, diverse block-wise missing-data patterns were introduced, and each approach was evaluated under five distinct missingness scenarios, illustrated in Fig. 1.

1. **Same Patterns**: Identical missing data patterns were applied to both the training and external testing datasets. This illustrates an ideal scenario in which the model faces identical missing data patterns during both training and testing. Success in this context suggests good learning under predictable data missingness, however, this might not capture the variability found in real applications.
2. **Light Generalization**: Most missing-data patterns present in the training dataset were also found in the external testing dataset, though not all. This simulates situations where the model encounters a small number of previously unseen patterns during testing. The aim is to assess whether the model can adapt to mildly unfamiliar missingness structures, reflecting real-world settings in which acquisition protocols or cohort characteristics introduce modest differences in modality availability.
3. **High Generalization**: Most of the missing data patterns in the training dataset were significantly different from those in the external testing dataset which is more challenging than the previous scenario. This reflects real situations where data collection methods vary, causing highly different missing data patterns. Performance under this condition is critical for evaluating an approach’s robustness and ability to generalize beyond the patterns observed during development.
4. **Missing Omic Simulation**: A specific omics layer was consistently present in the training dataset but absent from a portion of the testing dataset. This scenario simulates situations where certain tests are not performed in follow-up studies or validation cohorts. The goal is to evaluate whether the predictive model can effectively use the remaining omics layers to compensate for the missing data. Furthermore, this scenario also offers insight into the relative predictive contribution of each omics layer: sustained performance suggests redundancy across modalities, whereas substantial degradation may indicate the critical importance of the omitted omic.
5. **Training on Sparse Data**,** Testing on Rich Data**: The training dataset exhibited heavy missingness, whereas the testing dataset contained more available omics layers. In this scenario, the training dataset has a lot of missing data, while the testing dataset is more complete. This scenario flips the usual problem.The aim is to see if training with sparse data affects the model’s ability to use fully available data during testing. This setup is particularly relevant for longitudinal studies, where early-phase cohorts often have fewer modalities than later ones due to evolving technologies or funding differences.

### Data preprocessing and prediction model

A standardized preprocessing pipeline was used for all experimental scenarios to ensure consistency, reproducibility, and fair comparisons between models. To further enhance reliability, all experiments were repeated under 15 independent random seeds, and results were reported as mean ± standard deviation (SD) across these runs. To control for random variation within each seed, fixed random seeds were set across the operating system, NumPy, Python’s built-in random module, and TensorFlow. This multi-seed setup guaranteed both within-run reproducibility and across-run robustness, ensuring that any performance differences were due solely to the different approaches.

The preprocessing stage began with min-max normalization [24] of the feature values, bringing all omics-derived inputs into a comparable scale. This choice avoids inflation of large-valued features and supports stable neural network training across heterogeneous omics layers. The dataset included 705 patients, with 611 labeled as alive and 94 as deceased, leading to a significant class imbalance. To ensure a fair evaluation, the data were first split using a stratified hold-out scheme, with 5% of the samples reserved for testing and the remaining data used for training. This imbalance risked biased model learning, favoring the majority class. To address this, the training set was balanced by oversampling the minority class using random duplication to match the number of samples in the majority class. This produced a balanced training subset that improved sensitivity to the minority outcome and allowed the models to learn equally from both classes without sacrificing fairness or inflating the test-set performance.

Initially, several machine learning methods were tested, such as Random Forest [25], XGBoost [26], and LightGBM [27]. However, a deep learning model using Long Short-Term Memory (LSTM) [28] consistently showed the best performance for this dataset. Although LSTM networks are traditionally used for sequential data, their gated architecture can capture complex nonlinear interactions across high-dimensional omics features, which contributed to superior empirical performance. All experiments were implemented in Python using TensorFlow 2.19 and Scikit-learn. The final architecture included stacked LSTM layers with 64, 32, and 16 units, respectively, with each LSTM layer followed by a Dropout layer set to 0.2 to mitigate overfitting. A sigmoid activation function was used for the output layer. In addition to the earlier oversampling performed during preprocessing, focal loss [29] with parameters gamma = 2.0 and alpha = 0.25 was chosen as the objective function during model training to further emphasize the harder-to-classify samples. The Adam optimizer [30] with a learning rate of 0.001 was used for efficient gradient descent. The model was trained for 100 epochs with a batch size of 16, and early stopping was employed to prevent overfitting.

This carefully controlled and unified pipeline ensured that both intra-seed and inter-seed performance variations reflected true experimental differences rather than random effects or preprocessing inconsistencies. To facilitate full reproducibility, the exact software environment and package versions used in this study are provided in the accompanying repository.

### Experimental design and comparison

Four experiments were conducted to compare the performance of different approaches for handling block-wise missingness in multi-omics data. Three of these experiments are based on foundational ideas from existing frameworks in the literature, excluding their more complex enhancements to allow a fair comparison with the proposed baseline approach. The goal is to introduce a new fundamental strategy that, like the others, can later be extended or optimized.

Two of the experiments are available-case approaches, meaning they do not rely on imputation but instead work around the missingness structure. In Experiment 1, models are trained only for the specific missing patterns present in the training data. Although some studies do not address how the system should respond to previously unseen missing patterns in external data, this experiment addresses the issue by training a new model whenever such a pattern is encountered.

Experiment 2 takes a more exhaustive route by training models for all possible missingness patterns—specifically, 2^S^−1 models, where S is the number of omics types. This guarantees that every potential missing pattern during testing has a pre-trained model available.

Experiment 3 follows the imputation approach, applying imputation to both the training and testing datasets. To reduce randomness in the comparison, the same imputation technique which is K-Nearest Neighbors (KNN) was used, where applicable, in Experiment 4 as well.

Experiment 4 showcases our proposed approach, blending aspects of both available-case and Imputation-Based strategies. Like Experiment 1, models are trained only on the missing patterns found in the training set. However, if an unfamiliar missingness pattern emerges during testing, we apply imputation to transform the input into a compatible form, aiming to preserve biological integrity while maintaining computational efficiency.

Finally, for benchmarking purposes, the LSTM model was also applied to the uncurated dataset that contains no induced missing patterns. This baseline provides a reference for the model’s performance under ideal (fully observed) conditions.

To ensure that performance differences are attributable solely to the missing-data handling strategy, rather than to variations in the underlying prediction model, all four approaches were implemented using the same base classifier (LSTM). This deliberate design isolates the conceptual contribution of each strategy, allowing for a fair, controlled comparison.

Accordingly, the study focuses on comparing the core logic of missing-data handling rather than benchmarking against existing state-of-the-art integrative frameworks, which typically differ in model architecture, optimization objectives, and feature-level integration. Such differences would confound the interpretation of results by blending algorithmic improvements with the effects of the missing-data strategy itself.

All experiments were conducted using a fixed LSTM architecture and a frozen software environment to ensure reproducibility. Although it is not feasible to exhaustively test every possible combination of training and testing configurations across the vast landscape of block-wise missingness, the selected scenarios were intended to reflect a wide and clinically relevant range of patterns. Although alternative configurations may yield different comparative outcomes, the reported results offer strong empirical support for the effectiveness and generalizability of the proposed Hybrid Approach.

An overview of the key characteristics of all four experimental approaches is summarized in Table 1.

**Table 1:** Comparison of four experimental approaches for handling block-wise missingness in multi-omics data

Aspect	Experiment 1(Dynamic Available-Case approach)	Experiment 2 (Exhaustive Available-Case approach)	Experiment 3 (Full Imputation)	Experiment 4 (Proposed Approach)
Training Patterns	Only observed patterns in training data	All 2 S − 1 possible patterns	All training data imputed	Only observed patterns in training data
Testing Pattern Handling	Train new model for unseen patterns	Always covered (pre-trained models)	Imputed	Impute unseen patterns
Imputation Used	No	No	KNN	KNN (for unseen patterns)
Imputation Scope	None	None	Full dataset (train + test)	Test set only (as needed)
Adaptivity	Reactive (train-on-demand)	Exhaustive upfront	Static	Balanced (available-case + imputation)
Computational Load	Moderate	High	Moderate	Moderate

#### Available-case approaches: profile generation and data splitting 

As outlined in the previous section, Experiments 1, 2, and 4 share a common initial step, where the dataset is partitioned based on the availability of omics data. Each resulting subset contains only samples with complete data for a specific combination of omics layers. This process follows the general concept of profiles used in [17] where each sample is assigned a binary vector indicating the presence [1] or absence (0) of each omics layer. For instance, a profile vector of [1, 0, 0, 1] corresponds to a sample that contains gene expression (rs) and proteomics (pp) data, but lacks copy number (cn) and mutation (mu) data, reflecting the fixed order [rs, cn, mu, pp].

**Fig. 2:**
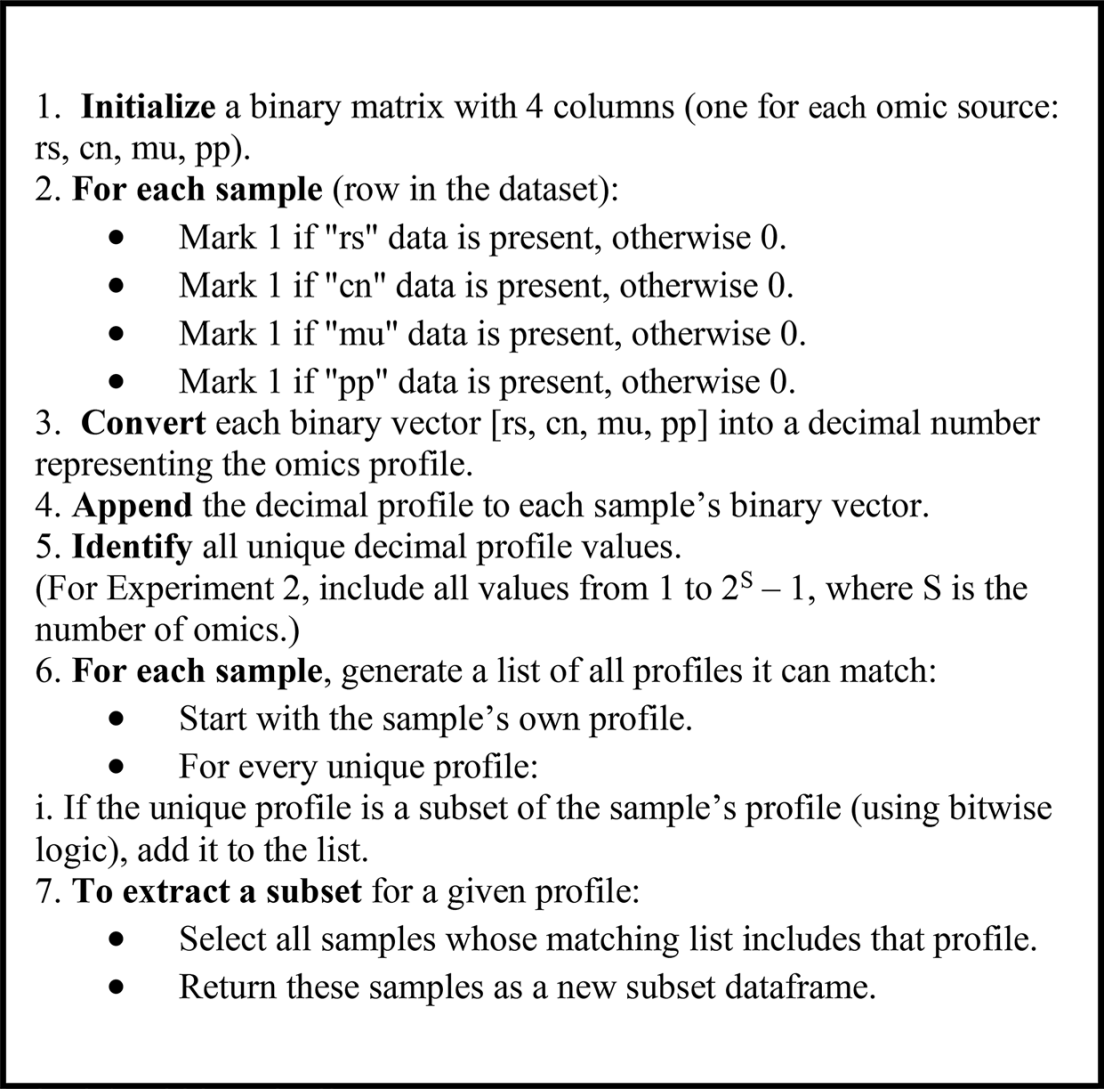
Pseudocode for creating subsets based on the profile concept

This profiling process is applied row by row to determine the missingness pattern of each sample. To ease data handling and grouping, each binary profile vector is converted to its corresponding decimal value. This enables efficient mapping of samples to subsets representing specific patterns of available omics data. In line with the approach described in [15], overlapping between subsets is permitted, meaning a single sample may belong to multiple subsets if its available omics satisfy the requirements of more than one profile. For example, a sample with the profile [1, 0, 1, 1] (decimal 11) contains data for rs, mu, and pp. It can therefore also be included in the subset corresponding to profile [1, 0, 1, 0] (decimal 10), as the required omics (rs and mu) are present. This strategy, which is described in Fig. 2, ensures maximum data utilization across subsets by leveraging every sample wherever it is applicable.

#### Imputation approach: KNN imputation method

As discussed in Sect.  3.4, Experiments 3 and 4 both incorporate imputation to varying extents. Experiment 3 relies entirely on imputed data, while Experiment 4 (our proposed approach) applies imputation selectively at test time, only when necessary. To ensure fairness, both use the same imputation strategy.

The imputation technique is based on a two-step K-Nearest Neighbors (KNN) approach [31]. First, the standard KNN imputation technique was applied using the KNNImputer from scikit-learn, with k set to 10 neighbors and distance-based weighting. Before imputation, all features were scaled using min-max normalization to ensure equal weighting during distance calculations. This step estimates missing values by computing a weighted average of the ten most similar samples, where similarity is measured using Euclidean distance across the available features. This approach effectively captures the local structure of the dataset.

After this initial imputation, a local refinement step was applied at test time to improve biological plausibility. For each incomplete test sample, refinement was performed within the subset of samples associated with a compatible target profile. Similarity was computed using Euclidean distance only over the features originally observed in the test sample, ensuring that distances were based exclusively on shared, non-missing information.

The nearest profile-compatible reference sample was then identified, and only the omics blocks missing in the test sample but required by the target model were replaced using the corresponding values from this reference. This procedure limits imputation to the minimum necessary features while preserving consistency with observed multi-omics patterns.

### Approaches for handling missing data patterns

**Fig. 3:**
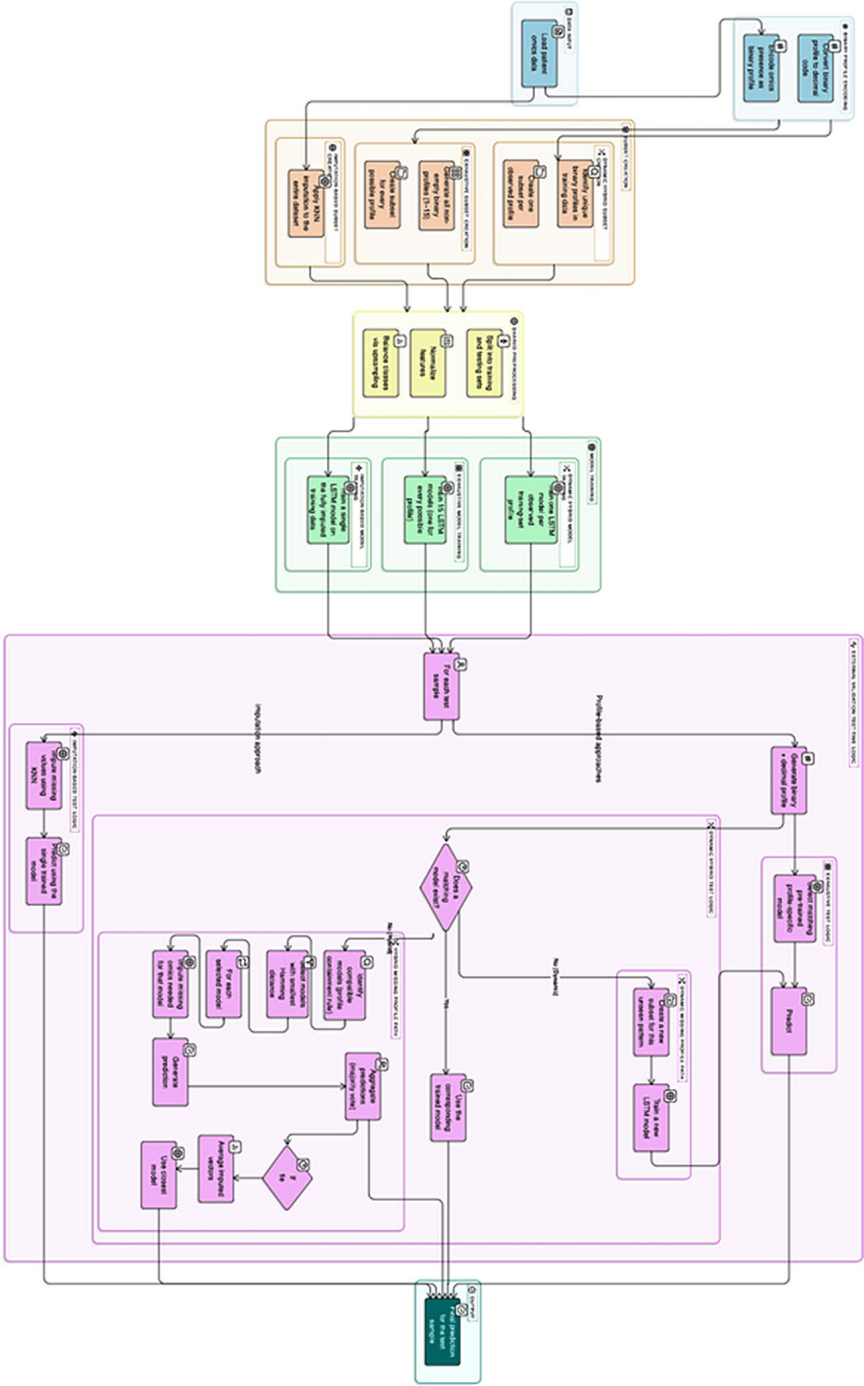
High-level overview of the four experimental workflows, showing shared components and method-specific branching for Dynamic Available-Case, Exhaustive Available-Case, Imputation-Based, and Hybrid Approaches. he Hybrid workflow incorporates majority voting with an optional tie-resolution step

Figure 3 presents a high-level overview of the four experimental pipelines, showing the steps that are shared across methods and indicating where the workflows diverge. The figure reflects the overall structure of the experiments, while the subsequent subsections (A–D) outline each approach in full detail.

#### Experiment 1: dynamic available-case approach

Experiment 1 employs an available-case approach leveraging the profile concept described in Sect.  3.4A. For each patient sample, the presence of data across omics sources is encoded as a binary profile vector, which is then converted into a decimal code to define subsets of samples sharing identical data availability patterns. The original dataset is partitioned into these subsets accordingly.

Each subset undergoes preprocessing as outlined in Sect.  3.3, including data balancing via upsampling to mitigate class imbalance and feature normalization to standardize scale. The balanced data is then split into training and testing sets. For each subset, an LSTM-based deep learning model is built and trained specifically on the data available for that profile.

During evaluation, an external validation set is used. Each test sample is profiled similarly to determine which model should be used for prediction. If a test sample presents a previously unseen profile (missing pattern), a new subset is dynamically created, a new model is trained, and the sample is predicted accordingly.

#### Experiment 2: exhaustive available-case approach

Similar to Experiment 1, Experiment 2 adopts a profile-based logic. However, in contrast to Experiment 1, which formed subsets only for the missing patterns observed in the training set, this approach creates a subset for every possible missing data pattern given the number of omics sources. A separate model is trained for each of these subsets.

With four-omics in the dataset, this results in 2^4^−1 = 15 distinct models, corresponding to all non-empty combinations of missing data patterns.

Each subset undergoes preprocessing consistent with Sect.  3.3, including class balancing via upsampling and feature normalization. These prepared subsets are then used to train LSTM-based models tailored to their respective data availability patterns.

During testing, each sample is profiled to determine its omics configuration, and the corresponding pre-trained model is selected for inference. Because all possible missing patterns were accounted for during training, this approach ensures complete coverage, eliminating the possibility of encountering unseen profiles.

#### Experiment 3: imputation-based approach

Experiment 3 applies a full-data imputation strategy. The entire training dataset is imputed using the two-step KNN technique detailed in Sect.  3.4B. This fully imputed dataset then undergoes preprocessing steps described in Sect.  3.3, including class balancing through upsampling and feature normalization. The processed data is split into training and testing sets, and a single LSTM model is trained on the entire dataset.

During evaluation, test samples are imputed individually using the same KNN-driven strategy to recover missing values. These completed samples are then passed to the trained model for prediction.

#### Experiment 4: the proposed Hybrid Approach

The proposed approach follows the same initial steps as Experiment 1, where a model is trained for each subset that matches a missing data pattern present in the training dataset. The same preprocessing pipeline and model architecture are applied across all experiments to ensure fairness in comparison. However, the key distinction emerges during evaluation using the external validation set. Each test sample is first profiled to identify its omics data availability pattern. If the profile exactly matches one of the pre-trained models, the corresponding LSTM model is used directly for prediction. Unlike Experiment 1, which trains a new model specifically for every unseen pattern in the external dataset, our approach leverages refined imputation to integrate the test sample into compatible pre-trained models.

To achieve this, the system searches for all candidate models with compatible profiles which are defined as those whose binary patterns “contain” the test sample’s pattern. That is, for every ‘1’ in the test sample’s profile, the candidate profile must also have a ‘1’ in the same position. For example, if a test sample has a profile of 0101 (indicating that only the second and fourth omics are available), then candidate models with profiles such as 1111, 0111, or 0101 would be considered compatible. The goal is to take advantage of all the already-available data in that test sample without discarding it. In rare cases where no superset-compatible model exists, which can occur in the sparse-training / rich-testing scenario, the framework falls back to subset-compatible models whose profiles are strict subsets of the test sample’s available omics. In this case, no imputation is performed, and predictions are based on the shared observed omics.

When imputation is applicable, the system selects the candidate model(s) with the smallest Hamming distance to the unseen profile, i.e., the most similar omics coverage, in order to minimize the imputation required for that sample. Since multiple models may have the same Hamming distance to the test profile, the test sample is imputed for each candidate model individually using the same two-step, distance-aware KNN imputation process employed in Experiment 3. In the default configuration, missing omics blocks are refined using values from a single most similar reference sample, which helps preserve biological coherence by ensuring that imputed blocks correspond to observed biological profiles. As an extension, we also implemented a weighted-average KNN variant in which missing features are estimated using a distance-weighted aggregation over multiple nearest neighbors. This alternative was evaluated across all experimental scenarios and random seeds. While it produced comparable performance in most settings, a modest improvement was observed only under the high-generalization scenario, accompanied by a small increase in test-time cost. Given the limited and scenario-specific gains, the single-nearest refinement strategy is retained as the default throughout the manuscript, and the averaged KNN variant is provided as an optional extension in the released code. This results in multiple imputed versions of the test sample, each aligned with a different candidate model. Each imputed sample is then passed to its corresponding LSTM model for prediction. The resulting predictions are aggregated using majority voting to produce the final prediction. An extended tie-breaking mechanism was also implemented for cases where the votes were evenly split. In this extension, the resulting imputations were aggregated into a consensus representation, and the consensus vector was mapped to the closest existing profile. The prediction of that nearest trained model was then used as the final output.

In practice, ties were extremely rare, and this extended tie-breaking mechanism did not yield measurable improvements in performance across any experiment or random seed. Therefore, all reported results use the primary majority-vote outcome. Nevertheless, the full tie-breaking extension is preserved in the codebase for methodological completeness and future development.

Alternative similarity measures such as Euclidean and Mahalanobis distances were also tested on a subset of validation samples. However, Hamming distance consistently yielded more stable alignment between profiles and better downstream classification accuracy, supporting its suitability for binary-pattern comparison in multi-omics contexts.

This dynamic strategy enables our approach to robustly handle unseen missing data patterns during testing by intelligently utilizing relevant, already-trained models. It avoids the need to retrain a new model for every unseen case, exhaustively train all possible models beforehand, or rely solely on integration techniques that might have unpredictable results and compromise the biological integrity of the dataset.

## Results

To assess the performance of the proposed approach, results from the complete (fully observed) dataset are used as a baseline, representing the best attainable performance under our current modeling pipeline. All reported performance values are expressed relative to this complete-data baseline. In some scenarios, performance exceeds 100% of the baseline, reflecting cases where the removal of noisy or weakly informative omics blocks leads to improved generalization, rather than “better-than-perfect” prediction. This evaluation follows a conceptual baseline comparison; accordingly, the goal is not to maximize accuracy through extensive hyperparameter tuning, but to provide a fair and controlled comparison of different missing-data handling strategies under identical modeling conditions. All approaches employ the same LSTM architecture, preprocessing pipeline, and experimental protocol.

A comprehensive experimental setup was designed involving four approaches: [1] Dynamic Available-Case approach [2], Exhaustive Available-Case approach [3], Imputation-Based approach, and [4] The Proposed Hybrid Approach. The experiments span five missing-data scenarios (as defined in Sect. 3.2), each simulating a distinct real-world form of incomplete multi-omics profile. Within each scenario, three training/testing pattern configurations were defined, resulting in 15 configurations per approach.

To ensure robustness and reproducibility, every configuration was repeated under 15 independent random seeds, accounting for stochastic factors such as weight initialization, data partitioning, and imputation randomness. Consequently, each approach was evaluated over 5 scenarios × 3 configurations × 15 seeds = 225 runs, totaling 900 evaluations across all approaches. The complete-data baseline was likewise retrained under the same 15 seeds to obtain a stable reference point. All performance results are reported as mean ± standard deviation (SD) across these 15 runs. Detailed per-seed results are provided in the Supplementary Material and accompanying repository.

The following metrics were used to evaluate each approach. These are derived from the confusion matrix, which consists of:

- TP (True Positives): Correctly predicted positive cases.
- TN (True Negatives): Correctly predicted negative cases.
- FP (False Positives): Negative cases incorrectly predicted as positive.
- FN (False Negatives): Positive cases incorrectly predicted as negative.

Each metric is calculated as follows:

6. Accuracy: Measures the overall proportion of correctly classified instances.

$$\:Accuracy=\:\frac{TP+TN}{TP+TN+FP+FN}$$

7. Balanced Accuracy: Averages the recall (sensitivity) for both classes to handle class imbalance. It reflects the method’s ability to avoid missing high-risk patients (deceased), which is essential for reliable prognostic modeling in oncology research.

$$\:Balanced\:Accuracy=\:\frac{1}{2}\:\times\:\:(\frac{TP}{TP+FN}+\frac{TN}{TN+FP})$$

8. F1 Score: The harmonic mean of precision and recall, emphasizing the balance between false positives and false negatives.

$$\:F1\:score=2\times\:\frac{Precision\:x\:Recall}{Precision+Recall}\:$$

9. Relative Performance: To assess the impact of missing data and the effectiveness of each approach, we express the performance metrics as a percentage of the baseline results obtained from the complete dataset. For each metric, the relative performance is calculated as:
  $$\eqalign{ & Relative\,Performance \cr & = {{Metric{\rm{}}\,of\,the{\rm{}}\,approach} \over {Baseline{\rm{}}\,Metric{\rm{}}}} \times 100 \cr}$$

This normalization allows a straightforward comparison across approaches, highlighting how close each approach gets to the ideal (complete data) scenario.

10. Training and Testing Time: Average time taken for training and testing (reported separately and combined), computed over three repetitions per configuration to smooth out system variability.

Finally, to assess whether the observed performance differences across methods were statistically meaningful, non-parametric Wilcoxon signed-rank tests were applied to the paired results obtained from the 15 random seeds. This test was chosen because it does not assume normality and is well-suited for bounded metrics such as accuracy, F1 score, and balanced accuracy. Each comparison was performed between the proposed Hybrid Approach and the other three approaches (Dynamic, Exhaustive, and Imputation-Based) using matched seed indices to ensure paired-sample consistency. All tests were conducted both overall and per-scenario, and the resulting *p*-values were adjusted using the Holm–Bonferroni correction to account for multiple comparisons. Statistical significance was established at an adjusted *p* < 0.05.

**Fig. 4:**
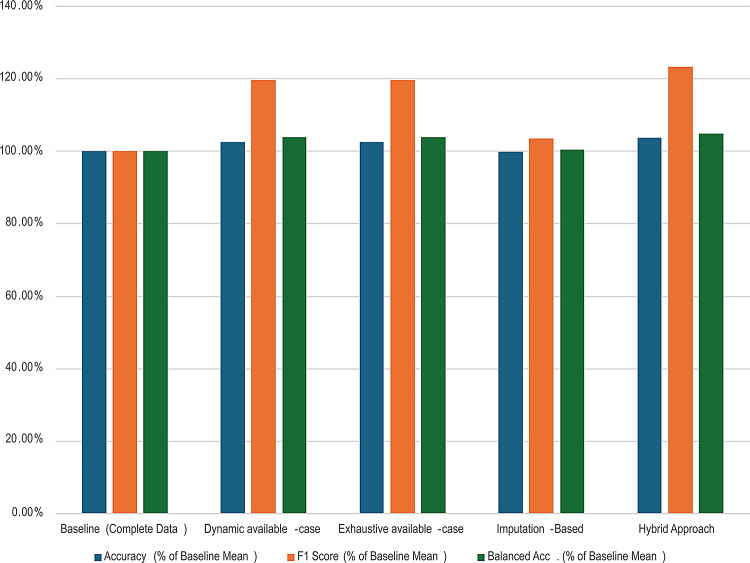
Performance metrics as relative percentages for the baseline and missing data approaches

**Fig. 5:**
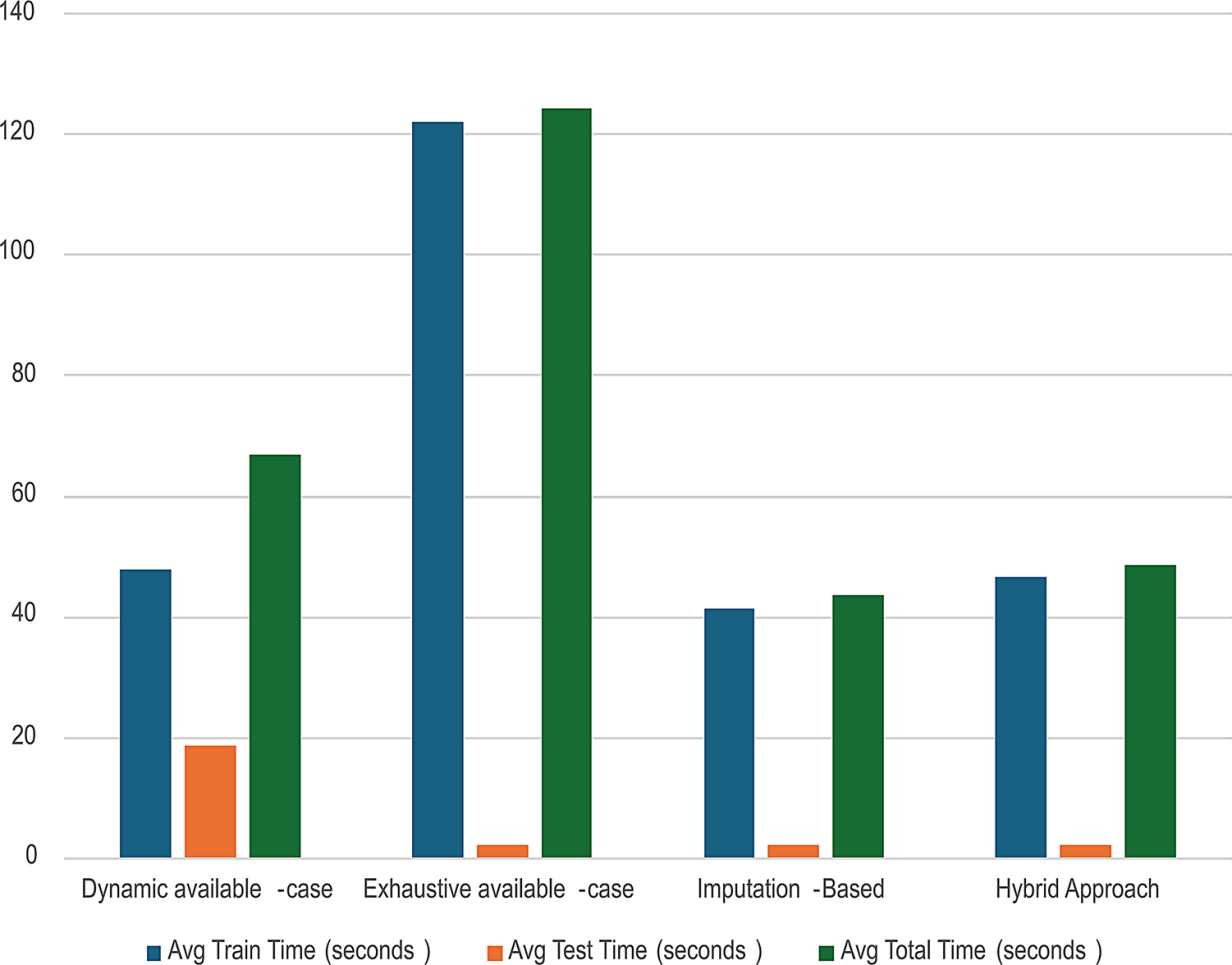
Average training, testing, and total runtime (seconds) per approach

Table 2 summarizes the overall evaluation results across the complete-data baseline and the four missing-data handling approaches. The reported metrics include mean ± standard deviation (SD) for accuracy, F1 score, and balanced accuracy, together with the average training, testing, and total runtimes. All performance values are also expressed as percentages relative to the baseline (complete data) to enable direct comparison. The adjusted p-values and corresponding effect sizes (r) are summarized in Table 3. Figure 4 visually compares the relative percentages of the performance metrics, while Fig. 5 illustrates the average training, testing, and overall times in seconds for each approach. The Hybrid Approach achieved the highest average relative accuracy, F1 score, and balanced accuracy among the evaluated missing-data strategies. Wilcoxon signed-rank tests further confirmed that these improvements were statistically significant over the Imputation-Based method in most scenarios, with medium-to-large effect sizes, while maintaining comparable or lower inference time.

**Table 2:** Summary of overall performance (mean ± SD) across 15 random seeds in five missingness scenarios

Approach	Accuracy	Accuracy (% of Baseline Mean)	F1 Score	F1 Score (% of Baseline Mean)	Balanced Accuracy	Balanced Acc. (% of Baseline Mean)	Avg Train Time (seconds)	Avg Test Time (seconds)	Avg Total Time (seconds)
Baseline (Complete Data)	0.82	100.00%	0.25	100.00%	0.57	100.00%	--	--	--
Dynamic Available-Case	0.84 ± 0.02	102.51%	0.31 ± 0.04	119.68%	0.59 ± 0.02	103.91%	47.97 ± 3.15	18.84 ± 13.03	66.81 ± 12.26
Exhaustive Available-Case	0.84 ± 0.02	102.51%	0.31 ± 0.04	119.68%	0.59 ± 0.02	103.91%	122.11 ± 9.96	2.23 ± 0.50	124.33 ± 9.54
Imputation-Based	0.82 ± 0.02	99.77%	0.26 ± 0.06	103.48%	0.57 ± 0.03	100.48%	41.44 ± 2.87	2.23 ± 0.24	43.67 ± 2.83
Hybrid Approach	0.85 ± 0.02	103.71%	0.32 ± 0.03	123.31%	0.60 ± 0.01	104.83%	46.59 ± 1.57	2.18 ± 0.32	48.77 ± 1.51

**Table 3:** Wilcoxon signed-rank test results for pairwise comparisons between the hybrid approach and other methods across 15 paired seeds

Comparison	Metric	*p*-value	Effect size (*r*)	Holm-adjusted *p*
Hybrid vs. Dynamic	Accuracy	6.99 × 10⁻⁹	0.30	1.40 × 10⁻⁸
—	Balanced Accuracy	9.37 × 10⁻⁴	0.17	1.87 × 10⁻³
—	F1 Score	4.76 × 10⁻³	0.15	9.53 × 10⁻³
—	Train Time	5.72 × 10⁻¹³	−0.37	5.72 × 10⁻¹³
—	Test Time	4.78 × 10⁻⁵⁷	−0.82	1.44 × 10⁻⁵⁶
Hybrid vs. Exhaustive	Accuracy	6.99 × 10⁻⁹	0.30	1.40 × 10⁻⁸
—	Balanced Accuracy	9.37 × 10⁻⁴	0.17	1.87 × 10⁻³
—	F1 Score	4.76 × 10⁻³	0.15	9.53 × 10⁻³
—	Train Time	3.32 × 10⁻⁶³	−0.87	9.97 × 10⁻⁶³
—	Test Time	0.7305	0.02	0.7305
Hybrid vs. Imputation	Accuracy	3.58 × 10⁻⁴⁵	0.73	1.07 × 10⁻⁴⁴
—	Balanced Accuracy	2.29 × 10⁻²⁵	0.54	6.88 × 10⁻²⁵
—	F1 Score	8.76 × 10⁻²⁵	0.53	2.63 × 10⁻²⁴
—	Train Time	1.23 × 10⁻³⁹	0.68	2.46 × 10⁻³⁹
—	Test Time	4.35 × 10⁻³	−0.15	8.69 × 10⁻³

Effect size (r) quantifies the magnitude of difference: small ≈ 0.1–0.3, medium ≈ 0.3–0.5, large > 0.5. A negative r indicates that the Hybrid Approach had lower values (e.g., shorter runtime) compared to the reference method, while a positive r means the Hybrid Approach had higher values for that metric (e.g., greater accuracy or F1 score)

### Same patterns scenario

In this scenario, each configuration uses the same set of missing data patterns for both training and testing, allowing us to examine how model performance varies when there is perfect alignment between the distribution of missing data in training and evaluation. Three configurations were designed, as detailed in Table 4. The average performance across all configurations, aggregated over 15 random seeds, is reported in Table 5. Since the Dynamic, Exhaustive, and Hybrid approaches yield identical accuracy, balanced accuracy, and F1 score values in this setting, no p-values are reported for these metrics. However, their training and testing times differ due to variations in computational strategy; therefore, only the timing comparisons are included in Table 6, alongside the full Hybrid versus Imputation-Based results.

**Table 4:** Training–testing pattern configurations for the Same-Pattern scenario, with distributions and rationale

Configuration	Training Patterns	Train Distribution	Testing Patterns	Test Distribution	Rationale
1	1101, 0111, 1011, 0110	[0.10, 0.10, 0.10, 0.10]	1101, 0111, 1011, 0110	[0.10, 0.10, 0.10, 0.10]	This setup uses a uniform training distribution across four multi-omics patterns, serving as a controlled baseline to assess performance when pattern exposure is evenly balanced between training and testing.
2	1110, 0110, 1011, 1001	[0.10, 0.10, 0.10, 0.10]	1110, 0110, 1011, 1001	[0.10, 0.10, 0.10, 0.10]	A fully balanced configuration, where all patterns are equally represented during both training and testing.
3	1110, 1011, 0001,0111	[0.10, 0.10, 0.05,0.10]	1110, 1011, 0001,0111	[0.10, 0.10, 0.025,0.10]	This configuration includes the rare and extreme pattern 0001, where only one omic is observed, testing each approach’s ability to generalize from sparse and unique cases.

**Table 5:** Average absolute (mean ± SD) and relative performance across all three configurations in the Same-Pattern scenario

Approach	Accuracy	Accuracy (% of Baseline Mean)	F1 Score	F1 Score (% of Baseline Mean)	Balanced Accuracy	Balanced Acc. (% of Baseline Mean)	Avg Train Time (seconds)	Avg Test Time (seconds)	Avg Total Time (seconds)
Dynamic Available-Case	0.84 ± 0.02	102.76%	0.29 ± 0.04	114.84%	0.59 ± 0.02	102.65%	51.94 ± 4.50	2.08 ± 0.17	54.02 ± 4.58
Exhaustive Available-Case	0.84 ± 0.02	102.76%	0.29 ± 0.04	114.84%	0.59 ± 0.02	102.65%	128.21 ± 5.17	2.30 ± 0.10	130.50 ± 5.24
Imputation-Based	0.81 ± 0.02	98.10%	0.23 ± 0.04	88.02%	0.55 ± 0.02	97.06%	40.58 ± 2.83	2.18 ± 0.13	42.76 ± 2.91
Hybrid Approach	0.84 ± 0.02	102.76%	0.29 ± 0.04	114.84%	0.59 ± 0.02	102.65%	48.06 ± 2.18	1.91 ± 0.08	49.97 ± 2.23

**Table 6:** Wilcoxon signed-rank test results comparing the hybrid vs. the other methods in the Same-Pattern scenario (15 paired seeds; Holm-adjusted p-values). Effect size r interpretation follows Table 3.

Comparison	Metric	*p*-value	Effect size (*r*)	Holm-adjusted *p*
Hybrid vs. Dynamic	Accuracy	—	—	7.72 × 10⁻³
—	Balanced Accuracy	—	—	1.61 × 10⁻²
—	F1 Score	—	—	9.41 × 10⁻³
—	Train Time	6.71 × 10⁻³	−0.70	6.71 × 10⁻³
—	Test Time	8.54 × 10⁻⁴	−0.86	1.31 × 10⁻³
Hybrid vs. Exhaustive	Accuracy	—	—	7.72 × 10⁻³
—	Balanced Accuracy	—	—	1.61 × 10⁻²
—	F1 Score	—	—	9.41 × 10⁻³
—	Train Time	6.10 × 10⁻⁵	−1.04	1.83 × 10⁻⁴
—	Test Time	6.10 × 10⁻⁵	−1.04	1.83 × 10⁻⁴
Hybrid vs. Imputation	Accuracy	2.57 × 10⁻³	0.78	7.72 × 10⁻³
—	Balanced Accuracy	5.38 × 10⁻³	0.72	1.61 × 10⁻²
—	F1 Score	3.14 × 10⁻³	0.76	9.41 × 10⁻³
—	Train Time	6.10 × 10⁻⁵	1.04	1.83 × 10⁻⁴
—	Test Time	6.53 × 10⁻⁴	−0.88	1.31 × 10⁻³

### Light generalization scenario

In this scenario, the goal is to assess how well the models can adapt to patterns that are only partially new, specifically those that share some similarities with the training data. Each configuration outlined in Table 7 introduces subtle differences between the training and testing sets that are similar enough to remain comparable, but distinct enough to challenge generalization. The average performance across all configurations, aggregated over 15 random seeds, is reported in Table 8. The corresponding Wilcoxon signed-rank test results, including Holm-adjusted p-values and effect sizes, are summarized in Table 9.

**Table 7:** Training–testing pattern configurations for the light generalization scenario, with distributions and rationale

Configuration	Training Patterns	Train Distribution	Testing Patterns	Test Distribution	Rationale
1	1011, 0111, 1101, 0110	[0.10, 0.10, 0.10, 0.10]	1101, 0101, 0111, 1011	[0.10, 0.10, 0.10, 0.10]	Adds one unseen-but-similar pattern (0101) to gauge responsiveness to modest novelty.
2	1110, 1101, 0111	[0.10, 0.10, 0.10]	1101, 0111, 1010,0101	[0.10, 0.10, 0.10,0.10]	Introduces two novel partial-overlap patterns (1010, 0101) posing a mild generalization challenge.
3	0101, 1011, 1100, 0001	[0.10, 0.10, 0.10,0.05]	0101, 1011, 1010	[0.10, 0.10, 0.20]	Tests generalization from sparse inputs via rare or extreme patterns.

**Table 8:** Average absolute (mean ± SD) and relative performance across all three configurations in the light generalization scenario

Approach	Accuracy	Accuracy (% of Baseline Mean)	F1 Score	F1 Score (% of Baseline Mean)	Balanced Accuracy	Balanced Acc. (% of Baseline Mean)	Avg Train Time (seconds)	Avg Test Time (seconds)	Avg Total Time (seconds)
Dynamic Available-Case	0.82 ± 0.02	100.51%	0.28 ± 0.06	108.51%	0.59 ± 0.03	102.40%	48.89 ± 3.18	13.06 ± 0.66	61.95 ± 3.55
Exhaustive Available-Case	0.82 ± 0.02	100.51%	0.28 ± 0.06	108.51%	0.59 ± 0.03	102.40%	129.31 ± 6.02	1.88 ± 0.07	131.20 ± 6.07
Imputation-Based	0.81 ± 0.03	98.32%	0.22 ± 0.05	86.63%	0.55 ± 0.02	96.66%	38.59 ± 2.61	2.38 ± 0.15	40.96 ± 2.74
Hybrid Approach	0.83 ± 0.01	101.46%	0.29 ± 0.05	111.72%	0.59 ± 0.02	103.21%	47.92 ± 5.94	2.09 ± 0.06	50.01 ± 5.96

**Table 9:** Wilcoxon signed-rank test results for the light generalization scenario (15 paired seeds; Holm-adjusted p-values). Effect size r interpretation follows Table 3

Comparison	Metric	*p*-value	Effect size (*r*)	Holm-adjusted *p*
Hybrid vs. Dynamic	Accuracy	4.60 × 10⁻³	0.73	1.38 × 10⁻²
—	Balanced Accuracy	1.33 × 10⁻²	0.64	2.67 × 10⁻²
—	F1 Score	4.65 × 10⁻³	0.73	1.39 × 10⁻²
—	Train Time	9.46 × 10⁻²	−0.43	9.46 × 10⁻²
—	Test Time	6.10 × 10⁻⁵	−1.04	1.83 × 10⁻⁴
Hybrid vs. Exhaustive	Accuracy	4.60 × 10⁻³	0.73	1.38 × 10⁻²
—	Balanced Accuracy	1.33 × 10⁻²	0.64	2.67 × 10⁻²
—	F1 Score	4.65 × 10⁻³	0.73	1.39 × 10⁻²
—	Train Time	6.10 × 10⁻⁵	−1.04	1.83 × 10⁻⁴
—	Test Time	6.10 × 10⁻⁵	1.04	1.83 × 10⁻⁴
Hybrid vs. Imputation	Accuracy	3.31 × 10⁻²	0.55	3.31 × 10⁻²
—	Balanced Accuracy	3.36 × 10⁻³	0.76	1.01 × 10⁻²
—	F1 Score	1.25 × 10⁻²	0.65	1.39 × 10⁻²
—	Train Time	6.10 × 10⁻⁵	1.04	1.83 × 10⁻⁴
—	Test Time	6.10 × 10⁻⁵	−1.04	1.83 × 10⁻⁴

### High generalization scenario

This scenario provides a greater challenge than the last one. The training set consists of various multi-omics patterns, while the test set introduces new combinations or partially overlapping patterns as shown in Table 10. This setup evaluates the models’ ability to generalize beyond the observed training distributions and accurately predict outcomes under unfamiliar missingness conditions. The average performance across all configurations, aggregated over 15 random seeds, is reported in Table 11 and the corresponding Wilcoxon signed-rank test results are reported in Table 12.

**Table 10:** Training–testing pattern configurations for the high generalization scenario, with distributions and rationale

Configuration	Training Patterns	Train Distribution	Testing Patterns	Test Distribution	Rationale
1	1110, 0111, 1011, 1101	[0.15, 0.15, 0.10, 0.10]	0001, 0010, 0100, 1010, 0011, 1001	[0.10, 0.05, 0.05, 0.10, 0.10, 0.10]	This configuration exposes the model to diverse multi-omics training patterns, then tests the model with a wide range of novel single- and dual-omics profiles in testing.
2	1001, 0111, 1010, 0001	[0.10, 0.10, 0.10, 0.10]	0011, 0010, 0101, 1110	[0.10, 0.10, 0.10, 0.10]	Tests generalization under full novelty by using non-overlapping test patterns absent from training.
3	1110, 1101, 1011, 0101	[0.10, 0.10, 0.10, 0.10]	1001, 1110, 0011, 1010, 1101, 0110	[0.10, 0.10, 0.10, 0.10, 0.10,0.10]	Blends familiar and novel patterns to challenge partial-transfer generalization while increasing distribution shift.

**Table 11:** Average absolute (mean ± SD) and relative performance across all three configurations in the high generalization scenario

Approach	Accuracy	Accuracy (% of Baseline Mean)	F1 Score	F1 Score (% of Baseline Mean)	Balanced Accuracy	Balanced Acc. (% of Baseline Mean)	Avg Train Time (seconds)	Avg Test Time (seconds)	Avg Total Time (seconds)
Dynamic Available-Case	0.82 ± 0.01	100.35%	0.26 ± 0.03	102.60%	0.57 ± 0.02	100.31%	47.82 ± 2.40	37.26 ± 3.41	85.08 ± 5.72
Exhaustive Available-Case	0.82 ± 0.01	100.35%	0.26 ± 0.03	102.60%	0.57 ± 0.02	100.31%	120.50 ± 5.67	1.96 ± 0.08	122.46 ± 5.74
Imputation-Based	0.83 ± 0.03	101.73%	0.32 ± 0.04	126.04%	0.60 ± 0.02	105.36%	42.14 ± 2.09	2.58 ± 0.07	44.72 ± 2.12
Hybrid Approach	0.84 ± 0.01	102.63%	0.31 ± 0.05	120.83%	0.59 ± 0.02	104.09%	45.66 ± 1.67	2.71 ± 0.08	48.38 ± 1.69

**Table 12:** Wilcoxon signed-rank test results for the high generalization scenario (15 paired seeds; Holm-adjusted p-values). Effect size r interpretation follows Table 3

Comparison	Metric	*p*-value	Effect size (*r*)	Holm-adjusted *p*
Hybrid vs. Dynamic	Accuracy	1.22 × 10⁻³	0.83	3.66 × 10⁻³
—	Balanced Accuracy	4.50 × 10⁻³	0.73	1.35 × 10⁻²
—	F1 Score	2.16 × 10⁻³	0.79	6.47 × 10⁻³
—	Train Time	1.53 × 10⁻³	−0.82	1.53 × 10⁻³
—	Test Time	6.10 × 10⁻⁵	−1.04	1.83 × 10⁻⁴
Hybrid vs. Exhaustive	Accuracy	1.22 × 10⁻³	0.83	3.66 × 10⁻³
—	Balanced Accuracy	4.50 × 10⁻³	0.73	1.35 × 10⁻²
—	F1 Score	2.16 × 10⁻³	0.79	6.47 × 10⁻³
—	Train Time	6.10 × 10⁻⁵	−1.04	1.83 × 10⁻⁴
—	Test Time	6.53 × 10⁻⁴	0.88	1.31 × 10⁻³
Hybrid vs. Imputation	Accuracy	8.20 × 10⁻¹	0.06	8.20 × 10⁻¹
—	Balanced Accuracy	3.46 × 10⁻¹	−0.24	3.46 × 10⁻¹
—	F1 Score	5.70 × 10⁻¹	−0.15	5.70 × 10⁻¹
—	Train Time	4.27 × 10⁻⁴	0.91	8.54 × 10⁻⁴
—	Test Time	4.51 × 10⁻³	0.73	4.51 × 10⁻³

### Missing omic simulation

In this setting we withhold an entire omics layer at test time. The three configurations rotate which source is missing as shown in Table 13. This scenario gauges how well each approach can leverage the remaining modalities, prevent dependence on a single omics block, and remain robust when entire data sources are missing for some samples, mirroring real-world platform failures. The average performance across all configurations, aggregated over 15 random seeds and the corresponding Wilcoxon signed-rank test results are reported in Tables 14 and 15.

**Table 13:** Training–testing pattern configurations for the missing omic simulation scenario, with distributions and rationale

Configuration	Training Patterns	Train Distribution	Testing Patterns	Test Distribution	Rationale
1	0011, 1011, 0111	[0.10, 0.10, 0.10]	1110, 0110, 1010	[0.05, 0.05, 0.05]	Trains only on profiles with pp = 1; tests on profiles where pp = 0.
2	1010, 1011, 0111, 0010	[0.10, 0.10, 0.10, 0.10]	1101, 1100	[0.15, 0.15]	Trains only on profiles with mu = 1; tests on profiles where mu = 0.
3	0100, 1110, 0101,1100	[0.10, 0.10, 0.10, 0.10]	1011, 1010, 1001, 1000	[0.10, 0.10, 0.05, 0.05]	Trains only on profiles with cn = 1; tests on profiles where cn = 0.

**Table 14:** Average absolute (mean ± SD) and relative performance across all three configurations in the missing omic simulation scenario

Approach	Accuracy	Accuracy (% of Baseline Mean)	F1 Score	F1 Score (% of Baseline Mean)	Balanced Accuracy	Balanced Acc. (% of Baseline Mean)	Avg Train Time (seconds)	Avg Test Time (seconds)	Avg Total Time (seconds)
Dynamic Available-Case	0.86 ± 0.02	104.98%	0.36 ± 0.03	139.76%	0.61 ± 0.01	107.40%	48.88 ± 3.64	24.82 ± 2.21	73.70 ± 5.65
Exhaustive Available-Case	0.86 ± 0.02	104.98%	0.36 ± 0.03	139.76%	0.61 ± 0.01	107.40%	125.76 ± 6.56	1.86 ± 0.07	127.61 ± 6.61
Imputation-Based	0.81 ± 0.02	98.19%	0.24 ± 0.04	92.01%	0.56 ± 0.02	97.58%	40.36 ± 2.50	1.94 ± 0.06	42.30 ± 2.53
Hybrid Approach	0.88 ± 0.02	106.63%	0.36 ± 0.05	142.10%	0.62 ± 0.02	108.13%	46.15 ± 1.32	2.28 ± 0.10	48.43 ± 1.34

**Table 15:** Wilcoxon signed-rank test results for the missing omic scenario (15 paired seeds; Holm-adjusted p-values). Effect size r interpretation follows Table 3

Comparison	Metric	*p*-value	Effect size (*r*)	Holm-adjusted *p*
Hybrid vs. Dynamic	Accuracy	3.24 × 10⁻³	0.76	6.47 × 10⁻³
—	Balanced Accuracy	0.151	0.37	0.302
—	F1 Score	0.140	0.38	0.280
—	Train Time	1.03 × 10⁻²	−0.66	1.03 × 10⁻²
—	Test Time	6.10 × 10⁻⁵	−1.04	1.83 × 10⁻⁴
Hybrid vs. Exhaustive	Accuracy	3.24 × 10⁻³	0.76	6.47 × 10⁻³
—	Balanced Accuracy	0.151	0.37	0.302
—	F1 Score	0.140	0.38	0.280
—	Train Time	6.10 × 10⁻⁵	−1.04	1.83 × 10⁻⁴
—	Test Time	6.10 × 10⁻⁵	1.04	1.83 × 10⁻⁴
Hybrid vs. Imputation	Accuracy	6.37 × 10⁻⁴	0.88	1.91 × 10⁻³
—	Balanced Accuracy	6.52 × 10⁻⁴	0.88	1.95 × 10⁻³
—	F1 Score	8.03 × 10⁻⁴	0.87	2.41 × 10⁻³
—	Train Time	1.22 × 10⁻⁴	0.99	2.44 × 10⁻⁴
—	Test Time	6.53 × 10⁻⁴	0.88	6.53 × 10⁻⁴

### Training on sparse data, testing on rich data scenario

In this scenario, each model is trained solely on high-sparsity profiles as illustrated in Table 15 and then challenged with richer test samples that include omics layers never encountered together during training. The aim is to determine whether an approach can make effective use of the newly available information at inference and to gauge how much training on incomplete data affects its final predictive performance. The average results across all configurations, aggregated over 15 random seeds, are reported in Table 17 and the corresponding Wilcoxon signed-rank test results are presented in Table 18.

**Table 16:** Training–testing pattern configurations for the sparse Train/Rich test scenario, with distributions and rationale

Configuration	Training Patterns	Train Distribution	Testing Patterns	Test Distribution	Rationale
1	0001, 0010, 1001, 1000	[0.10, 0.10, 0.10, 0.10]	1110, 0111	[0.15, 0.15]	Training covers one- and two-omic profiles; test probes generalization to dense three-omics combinations not seen during training.
2	0001, 0100, 1000, 0110	[0.10, 0.10, 0.10, 0.15]	1011, 1110, 0111	[0.15, 0.15, 0.15]	Training omits at least one key source each time; test samples reintroduce two new three-omic profiles.
3	0010, 0100, 0001, 1010	[0.10, 0.10, 0.10, 0.20]	1101, 1110	[0.20, 0.20]	Training covers sparse omics views; testing introduces novel multi-omic combinations, challenging the model to integrate across previously unlinked features.

**Table 17:** Average absolute (mean ± SD) and relative performance across all three configurations in the sparse Train/Rich test scenario

Approach	Accuracy	Accuracy (% of Baseline Mean)	F1 Score	F1 Score (% of Baseline Mean)	Balanced Accuracy	Balanced Acc. (% of Baseline Mean)	Avg Train Time (seconds)	Avg Test Time (seconds)	Avg Total Time (seconds)
Dynamic Available-Case	0.85 ± 0.01	104.12%	0.33 ± 0.05	129.25%	0.61 ± 0.02	106.12%	42.84 ± 2.53	17.48 ± 0.77	60.32 ± 3.13
Exhaustive Available-Case	0.85 ± 0.01	104.12%	0.33 ± 0.05	129.25%	0.61 ± 0.02	106.12%	105.60 ± 4.26	3.07 ± 0.15	108.67 ± 4.38
Imputation-Based	0.83 ± 0.02	100.57%	0.32 ± 0.05	124.31%	0.60 ± 0.03	105.33%	46.30 ± 1.41	2.10 ± 0.05	48.40 ± 1.43
Hybrid Approach	0.86 ± 0.02	104.93%	0.32 ± 0.05	126.48%	0.60 ± 0.02	105.83%	44.41 ± 11.45	1.93 ± 0.14	46.35 ± 11.46

**Table 18:** Wilcoxon signed-rank test results for the Rich–Sparse scenario (15 paired seeds; Holm-adjusted p-values). Effect size r interpretation follows Table 3

Comparison	Metric	*n* (seeds)	*p*-value	Effect size (*r*)	Holm-adjusted *p*
Hybrid vs. Dynamic	Accuracy	15	0.146	0.38	0.292
—	Balanced Accuracy	15	0.850	0.05	1
—	F1 Score	15	1	0	1
—	Train Time	15	0.303	−0.27	0.303
—	Test Time	15	6.10 × 10⁻⁵	−1.04	1.83 × 10⁻⁴
Hybrid vs. Exhaustive	Accuracy	15	0.146	0.38	0.292
—	Balanced Accuracy	15	0.850	0.05	1
—	F1 Score	15	1	0	1
—	Train Time	15	6.10 × 10⁻⁵	−1.04	1.83 × 10⁻⁴
—	Test Time	15	6.53 × 10⁻⁴	−0.88	1.31 × 10⁻³
Hybrid vs. Imputation	Accuracy	15	2.15 × 10⁻³	0.79	6.46 × 10⁻³
—	Balanced Accuracy	15	0.599	0.14	1
—	F1 Score	15	0.495	0.18	1
—	Train Time	15	0.035	−0.54	0.071
—	Test Time	15	1.06 × 10⁻²	−0.66	1.06 × 10⁻²

## Discussion

In real multi-omics pipelines and large-scale integrative analysis workflows, missing an entire omics layer is common due to budget, tissue scarcity, or sequencing failures, making the ability to handle block-wise missingness pivotal for real-world deployment. In this study, a Hybrid Approach was introduced to address block-wise missingness in multi-omics datasets and evaluated against two widely used strategies: available-case and Imputation-Based approaches, across five clinically meaningful and methodologically diverse scenarios. These scenarios were designed to reflect real-world patterns of data missingness, ranging from aligned train-test distributions to highly divergent and sparsely populated cases. The degree of missingness varied across scenarios and configurations, as indicated by the distributions presented in the corresponding tables, covering a wide range of realistic omics incompleteness patterns.

Despite the challenges posed by diverse missingness patterns, the Hybrid Approach consistently demonstrated strong and stable performance across all evaluated scenarios, outperforming the Imputation-Based method and matching or exceeding available-case strategies.

When the train and test distributions were closely aligned, all available-case approaches showed similar results, as expected. However, in more complex settings such as the Missing-Omic Simulation and Sparse-to-Rich scenarios, the Hybrid Approach consistently led across multiple metrics, including accuracy, F1 score, and balanced accuracy, while also maintaining low and predictable inference latency (~ 2.18 ± 0.32 s). In the Light- and High-Generalization conditions, it achieved either the top or second-best F1 scores, demonstrating a strong balance generalization across unseen simulated missingness patterns and computational efficiency.

Interestingly, in some cases, models exceeded the baseline set by complete data. This may be attributed to two main factors. First, block-wise missingness can function as an implicit form of regularization—eliminating noisy or weakly predictive omics layers reduces dimensionality and variance. Second, overlapping biological signals (e.g., TP53 status appearing across mutation, copy number, transcript, and protein layers) can lead to improved focus when noisy but redundant blocks are removed, thereby boosting minority-class recall and enhancing F1 and balanced accuracy. These findings highlight that more data is not always better when the additional omics layer contributes noise or redundancy rather than clinically meaningful signal.

Another important observation relates to the practical trade-off between training and testing times. The full-imputation strategy trained and predicted fastest, suiting large or time-critical datasets, but its performance wavers on novel patterns. Exhaustive Available-Case training is costly, yet inference is instantaneous, which is ideal when models are built rarely but queried often. Both Hybrid and Dynamic Available-Case approaches offered a more balanced solution. Notably, the Hybrid Approach combined low inference latency with consistent performance across diverse scenarios, making it a strong alternative to full imputation—offering comparable speed with better robustness. On the other hand, the Dynamic Available-Case approach showed higher variability in test-time performance, which could be problematic in settings where inference speed is critical.

From a data-integrity standpoint, preserving biological signal without distortion is essential. Although the Imputation-Based approach sometimes achieved strong predictive performance, its heavy reliance on inferred values—especially under block-wise missingness—produced noticeable run-to-run variability, raising concerns that imputation may insert biologically inaccurate signals. In contrast, available-case approaches preserve biological validity by using only genuinely observed data. While multiple imputation can yield more realistic values, applying it to every sample across a block-wise dataset is computationally expensive. The Hybrid Approach therefore incorporates imputation only in a targeted, test-time manner, enabling efficient alignment with trained models while preserving the interpretability and stability of the underlying biological signal. Further advances in imputation and available-case modeling are likely to enhance the Hybrid Approach’s biological reliability, as it builds on the strengths of both approaches.

While the absolute differences among methods may appear numerically small, these consistent gains across all 15 experimental configurations underline the Hybrid Approach’s robustness. It achieved the highest average relative balanced accuracy and F1 score while sustaining a low inference time, indicating that it provides the most efficient and reliable trade-off between performance and runtime for downstream prognostic modeling and patient-stratification tasks.

Overall, the statistical analysis confirms that the observed performance trends are consistent and reproducible across random seeds. Where differences were not statistically significant, they reflected small numerical gaps and normal seed variability rather than systematic advantages, reinforcing the robustness of the Hybrid Approach. Where accuracy is similar, the timing differences remain meaningful, Hybrid typically achieves lower test-time latency (negative r values). A few non-significant results, mainly in the Hybrid vs. Imputation comparison under the High-Generalization scenario, reflected small numerical gaps and normal seed variability rather than a consistent pattern. In this case, the Imputation-Based approach showed a marginally higher average accuracy, but the difference was not statistically significant (*p* = 0.82) and was accompanied by small, inconsistent effect sizes (*r* < 0.1), indicating no meaningful performance advantage. While the Imputation-Based method also achieved marginally faster test-time performance in this specific scenario, the Hybrid Approach maintained lower or comparable latency across the remaining scenarios, confirming its overall computational efficiency and stability. Overall, the results indicate that the observed trends are consistent and reproducible across the 15 seeds.

A major limitation of this study lies in the limited availability of fully observed four-omics datasets. Much of the current research is based on three-omics data, so a four-omics dataset was intentionally chosen to test missingness patterns across a broader set of omics layers. A fully observed dataset was essential as a reliable benchmark on which missingness could be systematically introduced. The chosen dataset, however, exhibits a significant class imbalance; this was mitigated with focal-loss optimization and careful stratified sampling. Despite these measures, class imbalance remains a concern and highlights the need for future work using naturally balanced datasets or more advanced methods to address imbalance.

Although the proposed approach demonstrates promising results, it reflects an extensible methodological framework rather than an optimized end-to-end system. Simplicity and clarity were deliberately prioritized, using straightforward model choices, basic data-balancing, and independent profile-specific handling without shared learning, to showcase the core idea. A practical challenge encountered in this work was the lack of publicly available implementations for many published methods, as noted in a recent review [5]. Consequently, the fundamental concepts common across these approaches were implemented rather than reproducing each system in full.

Finally, the proposed framework was evaluated on bulk multi-omics data and assumes sample-level profiles with block-wise missingness. Extending the approach to single-cell multi-omics data, which exhibit fundamentally different sparsity patterns and hierarchical structure, remains an important direction for future work. In addition, validation on independent datasets and real-world missingness patterns will be necessary to assess the broader applicability and generalizability of the proposed approach. Future work should also explore more balanced datasets, advanced imputation methodologies, and current techniques for cross-profile attribute sharing, all likely to improve overall predictive accuracy, biological validity, and enhance the utility of the Hybrid Approach for workflows where incomplete multi-omics profiles are routinely encountered.

## Conclusion

This study introduces a Hybrid Approach for handling block-wise missingness in multi-omics data. By combining the strengths of available-case modeling and Imputation-Based methods, the proposed framework offers a practical balance between biological integrity, computational efficiency, and predictive robustness.

Through extensive experiments on a complete four-omics dataset and under diverse simulated missingness scenarios, the Hybrid Approach demonstrated competitive accuracy and consistent performance across the missingness settings examined in this study. It consistently delivered stable results and often outperformed other approaches, particularly under generalization stress.

A major strength of the Hybrid Approach lies in its flexibility. It adapts to unseen missingness patterns by matching incomplete samples with existing models using lightweight imputation only when necessary. This strategy maximizes the utility of existing models, preserves biological integrity, eliminates the need for retraining, and maintains fast inference times.

By modularizing key components such as profile detection, model routing, and selective imputation, each part can work independently while contributing to the overall process. This modularity simplifies implementation and maintenance and allows for quick inference without retraining models for every new missing data pattern. The resulting framework is therefore well suited for large-scale multi-omics analysis pipelines that require reproducibility, stability, and predictable latency. Additionally, because models are reused and imputation is applied only when needed, the approach scales efficiently to large cohorts without significant computational cost.

Notably, it was observed that in several scenarios, the Hybrid and other approaches surpassed the complete-data baseline, suggesting that omitting weak or noisy omics blocks can enhance performance.

While this study does not exhaustively evaluate all missingness configurations, we have chosen scenarios that span a broad range of realistic and clinically relevant patterns. As such, they provide a strong basis for assessing the general applicability of the Hybrid Approach.

Despite limitations such as dataset constraints and class imbalance, the Hybrid Approach provides a reliable and computationally efficient solution to the challenge of working with incomplete multi-omics profiles. By enabling robust performance under substantial data incompleteness, the method contributes to the development of more stable and generalizable multi-omics predictive models. Future work may incorporate cross-profile learning, improved imputation strategies, and more advanced model architectures to further enhance its applicability and performance.

## Data Availability

The dataset used in this study is publicly available and was retrieved from Kaggle: [https://www.kaggle.com/datasets/samdemharter/brca-multiomics-tcga]. All code used in this study, together with per-seed experimental results and supplementary tables, is publicly available at: [https://github.com/esraahamdi/Hybrid-MultiOmics-Missingness.git]
